# Drug-induced skin reaction elicited by clindamycin and penicillin: one rash, but two culprits

**DOI:** 10.1186/2045-7022-4-S3-P139

**Published:** 2014-07-18

**Authors:** Wolfgang Pfützner, Markus Rose, Nils Nilles

**Affiliations:** 1 Philipps University Marburg, Department of Dermatology and Allergology, Germany

## Background

Cutaneous adverse drug reactions are often diagnosed by the appropriate diagnostic skin tests. However, since false-negative results are possible, one always has to consider performing drug provocation tests, especially if several drugs were taken in conjunction with a skin rash.

## Methods and results

A 75-year old male suffering from erysipelas received simultaneous treatment with oral clindamycin and i.v. penicillin G, which later was switched to oral penicillin. After eight days of antibiotic therapy an erythematous maculopapular exanthema developed on his trunk and extremities. Treatment was changed to cefuroxime, topical steroids initiated and the skin rash slowly vanished during the following days. Skin prick and intracutaneous tests were negative for penicillin, aminopenicillines, cefuroxime and clindamycine. Patch test revealed delayed type hypersensitivity against penicillin, but not cefuroxime or clindamycin. Thus, an allergic skin reaction against penicillin was assumed. To confirm tolerance of cefuroxime and clindamycin oral challenge tests were performed. However, while cefuroxime (maximal single dose 250mg/d; maximal cumulative dose 750mg/d) was well tolerated, on the third day of clindamycin application (maximal single dose 300mg/d; maximal cumulative dose 600mg/d) a maculopapular skin rash appeared, which was successfully treated with oral prednisolon. Since the positive patch test reaction could have simply resembled a penicillin sensitivity but not indicated a true allergic reaction, i.v. provocation with penicillin G was performed (maximal single dose 5 million IU/d; maximal cumulative dose 8 million IU/d). Interestingly, after two days a maculopapular rash developed, which was again efficiently treated with oral prednisolon.

## Conclusion

In summary, a delayed type drug reaction against both clindamycin and penicillin could be diagnosed, which became manifest as skin rash while both were taken at the same time. This case report underlines the value of provocation tests in diagnosing drug allergy, especially, when several drugs are potential culprits, and even if one drug has already been tested positive by skin tests.

